# Study protocol for “MDMA-assisted therapy as a treatment for major depressive disorder: A proof of principle study”

**DOI:** 10.3389/fpsyt.2022.954388

**Published:** 2022-10-26

**Authors:** Tor-Morten Kvam, Ivar W. Goksøyr, Lowan H. Stewart, Dimitris Repantis, Jan Ivar Røssberg, Ole A. Andreassen

**Affiliations:** ^1^Faculty of Medicine, University of Oslo, Oslo, Norway; ^2^Nordre Østfold DPS, Østfold Hospital Trust, Grålum, Norway; ^3^Awakn Clinics Oslo, Oslo, Norway; ^4^Department of Psychiatry and Neurosciences, Charité - Universitátsmedizin Berlin, Berlin, Germany; ^5^Oslo University Hospital, Oslo, Norway

**Keywords:** MDMA, major depressive disorder, clinical trial, depression, MDMA-assisted therapy, study protocol

## Abstract

**Background:**

Major depressive disorder (MDD) is a world-leading cause of disability. The available treatments are not effective in all patients, and there is a significant need for more effective treatment options. Here we present the protocol for an investigator-initiated and publicly funded trial of MDMA-assisted therapy (MDMA-AT) for MDD. This single-site, open-label study investigates the proof of principle and safety of MDMA-AT in participants with MDD and provides an initial impression of treatment effectiveness.

**Methods:**

A total of 12 participants [>18 years] with DSM-5 diagnosis of MDD will receive a flexible dose of MDMA in a therapeutic setting on two dosing days over a 4 week period preceded by three preparatory sessions. Each MDMA dosing session will be followed by three integration sessions. The primary outcome is change in MDD symptom severity, as measured by the mean change in MADRS scores from Baseline to 8 weeks after the second MDMA session. The secondary outcome is change in functional impairment, as evaluated by the mean change in Sheehan Disability Scale scores from Baseline to 8 weeks after the second MDMA session. Safety measures include vital signs, the incidence of Adverse Events and suicidality as measured by the Colombia-Suicide Severity Rating Scale.

**Discussion:**

This proof of principle trial will inform the development of fully powered clinical trials, optimize the protocol for the administration of MDMA-AT in participants with MDD and explore uncertainties including barriers to recruitment, retention and acceptability of MDMA-AT as a treatment for MDD.

**Clinical trial identification:**

EudraCT number 2021-000805-26.

## Background and rationale

Major depressive disorder (MDD) is a world-leading cause of disability, affecting more than 300 million people (1). Current treatment of MDD, including pharmacotherapy and psychotherapy are well-established and widely used (2, 3). However, the effect sizes are only small to moderate, the available treatment options are not beneficial for all MDD patients (3, 4), as many as 30% are regarded as treatment resistant (5), and antidepressant medications can have substantial adverse effects (3, 5). While the psychopharmacological revolution delivered a series of new drugs from 1950 to 1980's, few if any new agents have been developed during the last two decades (6, 7). The hope that advances in the neurosciences would enable highly effective forms of rational drug design have been disappointing (8). The primary treatments for MDD are psychotherapy and antidepressant medications (9) and large meta-analyses have concluded that the effect of these treatments to be comparable (10). The combination of pharmacotherapy and psychotherapy has shown to be the most effective treatment (11).

Decades after the restrictions of clinical research due to the international scheduling of 3,4-methylenedioxymethamphetamine (MDMA) and classical psychedelic drugs, there is now optimism associated with the treatment of mental disorders with these compounds in conjunction with psychotherapy. Randomized, controlled trials have demonstrated preliminary evidence for an antidepressant effect of the classical psychedelic psilocybin in participants with a life-threatening disease (12–14). A randomized, waiting list-controlled study found rapid and sustained antidepressant effect from psilocybin with a large effect size in participants with MDD (15). A randomized, double-blind study found psilocybin and escitalopram to be equally effective for MDD when analyzing the primary outcome measure. However, secondary outcome measures generally favored psilocybin (16). If MDMA and classical psychedelic drugs prove to be safe and effective in clinical trials, it will represent a new principle in the treatment of mental disorders and addiction: the facilitation of the psychotherapeutic process with an enduring effect and a rapid onset from one or a few doses as compared to the daily administration of currently available antidepressants.

MDMA acts primarily as a monoamine release and reuptake inhibitor, with a different mechanism of action than conventional antidepressant drugs. MDMA enhances synaptic levels of serotonin, and to a smaller extent norepinephrine and dopamine (17–20). Furthermore, MDMA affects neuroplasticity and increases the levels of oxytocin, vasopressin, cortisol and prolactin (21). Oxytocin is associated with prosocial behavior and increased trust and strengthening of the therapeutic alliance (22), supporting the use of MDMA in combination with therapy (23). Many MDD patients have excess activity of the hypothalamic-pituitary-adrenal cortex (HPA) axis, potentially causing neurobiological changes including hippocampal atrophy (24). The indirect effects of MDMA on neurohormone release may help regulate the HPA axis, which is a central psychopathological feature of MDD (25).

MDMA-assisted therapy (MDMA-AT) is a novel alternative approach for treatment of mental disorders (26). In this treatment model, MDMA is regarded as a catalyst for the psychotherapeutic process (27), and is administered with therapy in 2 or 3 monthly 8-h sessions, preceded by preparatory and followed by integrative therapy sessions (28). Six phase 2 trials and one phase 3 trial have demonstrated significant effects of MDMA-AT for post-traumatic stress disorder (PTSD) compared with placebo, with large effect sizes (29, 30). In 2017, the Food and Drug Administration (FDA) designated MDMA-assisted psychotherapy of PTSD a “breakthrough therapy” (26). MDMA-AT has shown preliminary positive effects in other indications as well (31–33), however it has not been investigated in the treatment of MDD until now.

In the following text, we will provide the rationale for an MDMA-AT trial for MDD. Our design is based on the first study of psilocybin for treatment-resistant depression, which was an open label trial in a sample of 12 participants (34), and a MDMA-AT trial for PTSD, for which the study team was a site (35).

Case reports from the 1970's and 1980's described therapeutic benefits in a variety of psychological disorders, including depression, although no clinical trials were conducted at that time before MDMA was scheduled as an illegal drug (36). Greer and Tolbert summarized the potential benefits in 29 patients that received MDMA as an adjunct to psychotherapy (27). Nine subjects with a psychiatric diagnosis reported significant relief, and all four patients with a depressive disorder reported improvement (27).

MDMA-AT has shown preliminary but promising results in other indications, namely in the treatment of social anxiety in autistic adults (31), anxiety associated with a life threatening illness (32) and alcohol use disorder (33). Although these results need to be confirmed in larger, randomized trials, they indicate transdiagnostic benefits from MDMA-AT, which in turn could be extended to MDD.

Significant and immediate antidepressant effects of MDMA-AT have been demonstrated in two published phase 2 PTSD trials (37, 38). In the pooled analysis of all six phase 2 trials, there was a trend toward significance, whereas the phase 3 trial showed also an antidepressant effect (28, 30). A significant proportion of MDD patients have experienced traumatic events earlier in life, and childhood trauma is associated with depression in adulthood (39). To the extent that unprocessed traumatic events is a causative factor in some patients with depression, this would suggest that the benefit from MDMA-AT observed in the PTSD trials may also apply to MDD trials (40).

MDD often relapses after initial response or remission (41). The PTSD studies show not only a lasting, but also increased positive effect on PTSD symptoms at the long-term follow-up one year after MDMA-AT (28, 29). If the results from the PTSD trials are applicable to MDD, sustained efficacy after a few doses might prevent relapse and could also be beneficial in comparison to the daily administration of current available medication and the associated adverse effects.

Although speculative, indirect evidence from PTSD studies makes it possible to hypothesize a similar impact on MDD, including improved self-knowledge, sleep regulation, mentalization skills, coping strategies, emotion regulation, and cognitive insights (28). As MDMA temporarily increases interpersonal trust, while also reducing avoidance and psychological defenses, it might be helpful in the processing of memories and emotions in MDD same as in PTSD (42). Furthermore, MDMA-AT allows for an experience of safety, trust and collaboration with the therapists (43). Overall, MDMA-AT holds promise as a catalyst to the therapeutic process and enhancer of the therapeutic alliance.

Although recreational use of MDMA sometimes is associated with low mood in the days following ingestion, this is less of a concern when MDMA is administered clinically (44). There are several reports on adverse events from recreational use of MDMA, however these findings are based on studies with sub-optimal methodology (45). This includes the inclusion of polydrug users, the use of higher doses than in a clinical setting and that the drugs taken contain other substances in addition to or instead of MDMA. Pure MDMA has been well-tolerated in controlled studies in humans (46). Close to 200 participants have received MDMA-AT for PTSD in phase 2 and 3 trials (28). The US FDA has declared MDMA to have a satisfactory safety profile for clinical trials to be conducted (26). Nevertheless, clinical trials with MDMA must be careful to address potential adverse effects and risk of long-term harmful effects.

The primary objective of this pilot study is to explore the proof of principle and safety of open-label MDMA-AT with a flexible dose of MDMA in participants with MDD, and provide an initial assessment of treatment effectiveness. This proof of principle study will inform the development of fully powered RCTs, optimize the protocol for the administration of MDMA-AT in participants with MDD and explore uncertainties including barriers to recruitment, retention and acceptability.

## Methods and analysis

### Design

This single-site, open-label study investigates the proof of principle and safety of MDMA-AT in 12 participants diagnosed with MDD and provides an initial assessment of potential treatment effectiveness.

A study protocol of MDMA-AT for PTSD (35), from which the study team has experience as a study site, informed the development of the present study protocol. In the treatment period, MDMA is given on two dosing days ~1 month apart. A supplemental half-dose of MDMA will be provided to extend the effects unless tolerability issues. A total of nine 90-min non-drug preparatory and integrative psychotherapy sessions will precede and follow the MDMA dosing sessions. Dosing sessions are followed by an overnight stay.

As in (35), for each participant, the study will consist of:

° Screening period: phone screen, informed consent, eligibility assessment, and enrollment of eligible participants.
° Preparatory period with enrollment confirmation: medication tapering, preparatory sessions and baseline assessments.
° Treatment period: two monthly dosing sessions and associated integrative sessions over ~8 weeks. The aims of the integration sessions are to continue the exploration of emotions and memories and to apply any new insights and experiences into daily life.
° Follow-up period and study termination: 4 weeks with no study visits, followed by outcome measures and study termination visit.

### Recruitment and participant population

We will recruit adult men and women, 18 years of age and older, with at least moderate depression at time of enrollment. Participants with depression are defined as those who meet the Diagnostic and Statistical Manual of Mental Disorders, 5th Edition (DSM-5) (47) diagnostic criteria for single or recurrent episode of MDD without psychotic features, with a duration of more than 12 weeks and < 2 years at time of enrollment. Participants will be recruited through referrals from general practitioners, psychiatrists, and psychotherapists. During screening, the participant may be asked to taper concomitant medications, if needed. The key inclusion and exclusion criteria are shown in Table 1. Key inclusion and exclusion criteria.

**Table 1:** Key inclusion and exclusion criteria.

**Inclusion criteria [informed by (35)]**
• At Screening, meet DSM-5 criteria for current MDD with a symptom duration of more than 12 weeks and less than 2 years • At Baseline, have at least moderate MDD per MADRS and symptoms in the last month constituting a MADRS Total Severity Score of 20 or greater • ≧18 years of age • Sufficient competency in the Norwegian language • Capacity to provide consent • Agreement to provide a support person who is willing and able to be reached by the investigators in the event of a participant becoming suicidal or unreachable • Agreement to follow specific lifestyle modifications **Exclusion criteria**
• Electroconvulsive Therapy (ECT) or ketamine within 12 weeks of enrollment • Current or previously diagnosed psychotic disorder, significant history of mania, or evidence of a personality disorder • Current eating disorder with active purging • Current moderate or severe alcohol or cannabis use disorder within the 12 months prior to enrollment • Active illicit (other than cannabis) or prescription drug substance use disorder at any severity within 12 months prior to enrollment • Current serious suicide risk, as determined through psychiatric interview, responses to C-SSRS, and clinical judgment of the investigator will be excluded; however, history of suicide attempts is not an exclusion • The use of any psychoactive medication not approved by the research team from Baseline through Study Termination (with the exception of gabapentin or certain opiates for pain control) • Positive pregnancy test or breastfeeding. Unable to agree to the use of highly effective contraception methods • History of any medical condition that could make receiving a sympathomimetic drug harmful because of increases in blood pressure and heart rate. This includes, but is not limited to, a history of myocardial infarction, cerebrovascular accident, or aneurysm. Mild, stable chronic medical problems (diabetes mellitus type 2, for example) are allowed in the study if the symptoms from that disease are not being confused with side effects from MDMA or interfering with study participation • Uncontrolled hypertension. Participants with well-controlled hypertension might be enrolled if acceptable cardiovascular risk (family history, smoking, lipid levels, body weight, level of physical activity) and no signs of ongoing cardiovascular or cerebrovascular disease from history, physical exam or ECG supplemented by echocardiography or nuclear imaging, and carotid ultrasound • History of ventricular arrhythmia at any time, other than occasional premature ventricular contractions (PVCs) in the absence of ischemic heart disease • Have a marked Baseline prolongation of QT/QTc interval e.g., repeated demonstration of a QTc interval >450 milliseconds [ms] in males and >460 ms in females (corrected by Fridericia's formula) • Have a history of additional risk factors for Torsade de pointes (e.g., heart failure, hypokalemia, family history of Long QT Syndrome) • Symptomatic liver disease or significant liver enzyme elevation • History of clinically significant hyponatremia or hyperthermia • Weight < 48 kilograms (kg)

As this is a feasibility study, we are interested in obtaining a preliminary impression of how MDD patients can be recruited to an MDMA-AT study, and to what extent the patient remains in the study as a proxy for the acceptability of the treatment. We will report the number of potential participants that were phone screened, as well as the number of participants that were considered as screen failures (not eligible after signed informed consent form and therefore not enrolled in the study), pre-dosing early terminations (initially enrolled, but deemed ineligible before the first MDMA session), post-dosing early terminations (Participants who choose to stop treatment, but who attend study visits and participate in outcome assessments), dropout (Participants who withdraw their consent and do not want to contribute to further study visits or measurements), and lost to follow-up (Participants who do not show up for study visits and who are unable to be contacted by the site staff).

### Study visits

**Figure 1:**
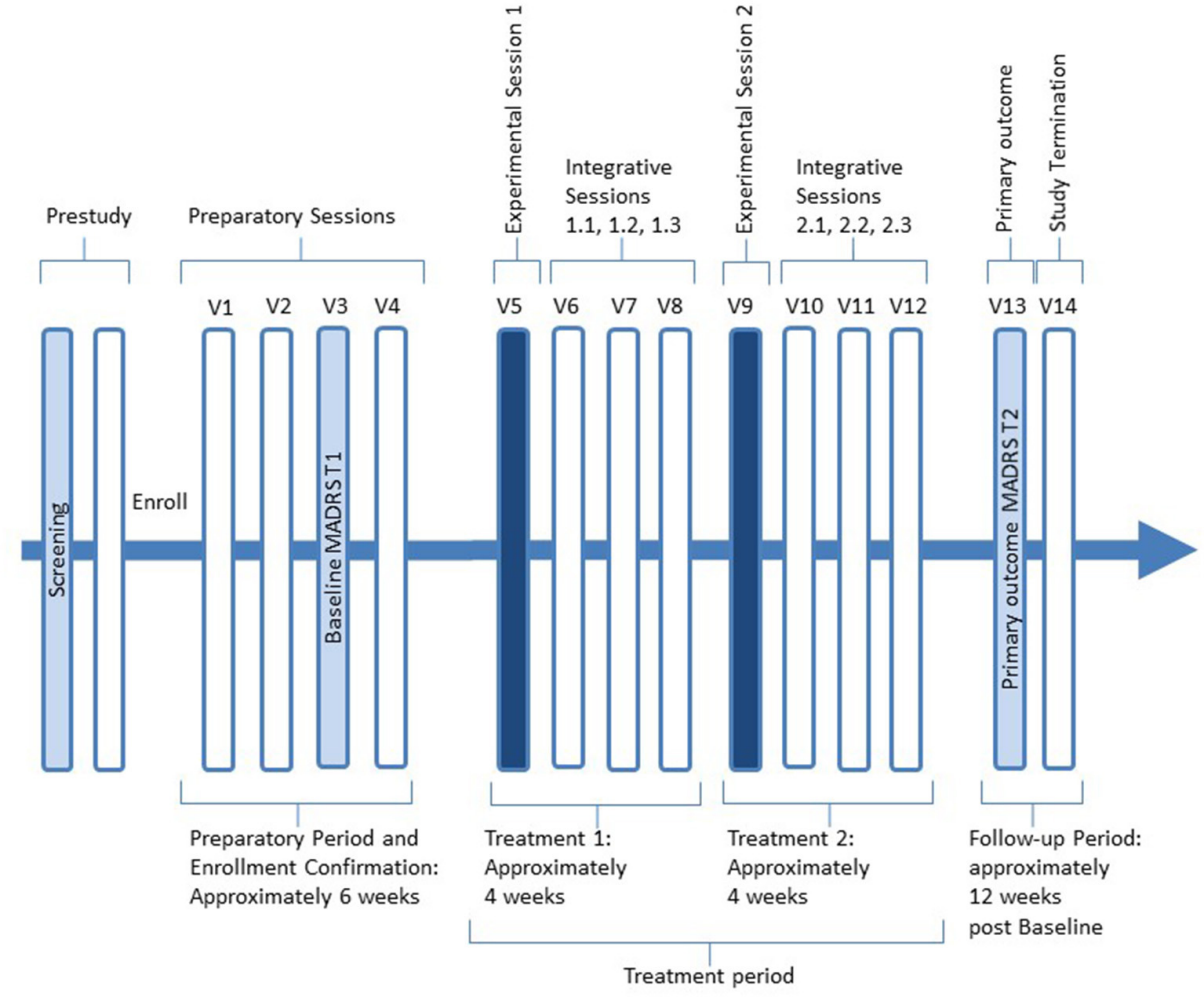
Study structure overview.

The Study Structure Overview is shown in Figure 1. Full screening may take 2 to 5 weeks after phone screening. Enrollment takes place at the completion of Screening. Safety information and measures will be collected up to 1 month after the participant's last therapy session during the Follow-Up Period. We will recruit actively up to enrollment of 12 participants. The Preparatory Period begins at enrollment and can be as short as 2 weeks. If the participant uses psychotropic medication, the preparation period can be up to 8 weeks to ensure adequate tapering until discontinuation plus one week of stabilization without medication.

The preparatory period ends at enrollment confirmation and thus the start of the treatment period. The treatment period lasts approximately 8 weeks and includes two dosing sessions 3-5 weeks apart and three non-drug integrative sessions after each of the two. After the final Integrative Session 2.3 (Visit 12), participants will enter the follow-up period with no study visits for ~4weeks, followed by the Primary Outcome MADRS T2 (Visit 13). The study procedures are presented in Table 2.

**Table 2:** Time and events – study procedures [informed by (35)].

**Time and events – study procedures**	**Screening period (2 to 6 weeks)**			**Preparatory period w/enrollment confirmation** **(2 to 11 weeks)**			
	**Screening**		**Enrollment**	**Preparatory**		**Baseline MADRS T1**	**Baseline & enrollment confirmation**
**Visit**	**Phone screening**	**Screening**	**V0**	**V1**	**V2**	**V3**	**V4**
**Visit description**	**Phone calls**	**In-person visits & labs**	**Enrollment**	**Prep 1**	**Prep 2**	**MADRS T1 assessment at site**	**Prep 3 & enrollment confirmation**
Initial phone screen	✓						
Informed consent	Send Copy	✓					
Follow-up phone screen	✓						
Assess eligibility	✓	✓	✓	✓	✓		✓
Medical/psychiatric history	✓^A^	✓		✓	✓		✓
Past/current medication & adherence	✓	✓		✓	✓		✓
Weight, resting vitals		✓					
Physical exam		✓					
ECG & rhythm strip^H^		✓					
Clinical lab tests		✓					
Drug screen		✓					✓
Pregnancy screen		✓					✓
Enter participant in eCRF		✓					
Record				✓	✓	✓	✓
Medication taper			✓	✓	✓		
Study enrollment			✓				✓ Confirmed
All AEs^B^			✓	✓	✓		✓
90-min preparatory session				✓	✓		✓
Phone call follow-up^C^					✓		
**Time and events – study procedures (continued)**	**Treatment period** ~**8 weeks (**+**/-3 weeks)**								**Follow-up period & study termination (**~**4 weeks)** **13 weeks (**+**/-3 weeks) post baseline**	
	**Treatment 1**				**Treatment 2**				**Outcome**	**Study Termination**
**Visit**	**V5**	**V6**	**V7**	**V8**	**V9**	**V10**	**V11**	**V12**	**V13**	**V14**
**Visit description**	**Exp. 1**	**Int 1.1**	**Int. 1.2**	**Int 1.3**	**Exp. 2**	**Int. 2.1**	**Int 2.2**	**Int 2.3**	**MADRS T2 assessment at site**	**Study termination**
Past/current medication and adherence	✓	✓	✓	✓	✓	✓	✓	✓		✓
Drug screen	✓				✓					
Pregnancy screen	✓				✓					
Record	✓	✓	✓	✓	✓	✓	✓	✓	✓	
All AEs^D^	✓	✓	✓	✓	✓	✓	✓	✓		✓
Administer IP	✓				✓					
8-hour Exp. Session	✓				✓					
BP, Pulse, Temperature^E^	✓				✓					✓^F^
Overnight Stay	✓				✓					
90-min Integrative Session		✓	✓	✓		✓	✓	✓		
Phone Call Follow-up^G^		✓				✓				
Weight										✓

^A^At Screening, collect data on previous hospitalizations, healthcare utilization and economic productivity. Request participants to obtain medical/psychiatric records to bring to the in-person screening.

^B^All Adverse Events (AEs) includes collecting Serious Adverse Events, AEs of Special Interest, AEs of Psychiatric Status, AEs requiring medical advice or attention, AEs that indicate withdrawal of a participant, and all other AEs.

^C^If needed, call participant to confirm medication tapering and stabilization is complete prior to Visit 3.

^D^All Adverse Events (AEs) includes collecting Serious Adverse Events, AEs of Special Interest, AEs of Psychiatric Status, AEs requiring medical advice or attention, AEs that indicate withdrawal of a participant, and all other AEs.

^E^During Dosing Sessions, vitals are measured before Investigational Product administration, immediately before the supplemental dose is administered (or would be, if supplemental dose not given), and approximately 8 h after initial dose, and as needed.

^F^At Study Termination, only blood pressure needs to be measured.

^G^4 days of phone call follow-up: Day 2 and 7 after the Dosing Session, with two additional calls in between.

^H^We do not repeat the ECGT during the MDMA dosing sessions, since we will avoid possible interference with the therapy facilitated by MDMA.

### MDMA-assisted therapy

Overall, the goal of the therapy is to reduce MDD symptoms and improve the function of the participant. The study therapists have been trained in an MDMA-assisted therapy training program in a previous clinical trial.

During the preparatory period, the therapists begin establishing an effective therapeutic alliance, addressing any questions or concerns the participant may have, as well as familiarizing them with the therapeutic approach.

MDMA-AT is largely inner-directive, i.e., allows each participant's experience to unfold spontaneously without providing a specific direction or advice about the content of the experience. Important elements are creating a setting of safety and support, empathic listening, inviting the participant to explore emerging material and make suggestions instead of having a set agenda (48). In addition, support for surfacing challenging material is provided in a way that does not get in the way of the participant's emerging experience. The MDMA sessions will take place in a private, aesthetically pleasing and quiet room with flexible ambient temperature. The participant can either lie down on the bed or sit up with support from pillows and alternate between introspection and interaction with the therapists. The use of eyeshades and headphones is suggested while the participant “goes inside.”

The goals of the integrative sessions are to help the participant continue to process emerging material and provide support if any challenges arise during this process, and invite the participant to apply any new insights, perspective and attitudes to daily life.

### Outcome measures

#### Primary outcome measure

The primary outcome measure will capture the change in MDD symptom severity, as measured by the mean change in MADRS scores from Visit 3 (Baseline) to Visit 13 (approximately 12 weeks post Baseline) (49).

#### Secondary outcome measure

The secondary outcome measure will capture the change in functional impairment, as measured by the mean change in the clinician-rated Sheehan Disability Scale (SDS) scores from Visit 3 (Baseline) to Visit 13 (< 12 weeks post Baseline) (50).

#### Safety outcome measures

The C-SSRS is a clinician-administered measure of suicidal behavior designed to detect any suicidal thoughts or behaviors during a clinical trial (51). It consists of a Baseline/Screening version and a Since Last Visit version. The Baseline/Screening version will be administered during screening, and the Since Last Visit version will be used at all subsequent visits. The C-SSRS assesses suicidal ideation, ideation intensity, and behavior.

Safety outcome measures that will evaluate the safety of MDMA-AT in a clinical practice setting are shown in Table 3 [informed by (35)].

**Table 3:** Safety outcome measures.

1. Assess incidence of AEs during Dosing Sessions that may be indicative of a medical complication of the Investigational Product (IP), such as clinical signs and symptoms of chest pain, shortness of breath, or neurological symptoms or any other signs or symptoms that prompt additional vital sign measurements
2. Assess incidence of AEs by severity
3. Assess incidence of Treatment Emergent AEs (TEAEs) by severity
4. Assess incidence of TEAEs by severity taken during a Dosing Session and 2 days after IP administration
5. Assess incidence of AESIs, defined as AEs specified in the protocol related to cardiac function, suicide risk, and abuse liability
6. Assess incidence of AEs by severity categorized as leading to discontinuation of IP, resulting in death or hospitalization, and continuing at Study Termination
7. Assess incidence of SAEs
8. Assess incidence of psychiatric concomitant medications taken during a Dosing Session and 2 days after IP administration
9. Assess incidence of any psychiatric concomitant medications taken during the Treatment Period
10. Assess incidence of serious suicidal ideation and positive suicidal behavior assessed with the Columbia Suicide Severity Rating Scale (C-SSRS)
11. Assess mean changes in blood pressure, heart rate, and body temperature from pre-IP administration to the end of each Dosing Session

#### Exploratory outcome measures

The exploratory outcome measures will explore self-reported depression symptom severity [Beck Depression Inventory II (BDI-II)] (52), quality of life [EuroQol Five Dimensions – Five Levels Questionnaire (EQ-5D-5L)] (53), wellbeing [Warwick Edinburgh Mental Well-being Scale (WEMWBS)] (54), self-compassion [the Self-Compassion Scale (SCS)] (55), alexithymia [the Toronto Alexithymia Scale (TAS-20)] (56), alcohol use [Alcohol Use Disorders Identification Test (AUDIT)] (57) and substance use [Drug Use Disorders Identification Test (DUDIT)] (58). These outcome measures will be assessed during screening and/or at the Baseline visit, and repeated at the Study Termination visit. The BDI-II will also be assessed at visits 8 and 12.

### Study governance and monitoring

The sponsor-investigator will ensure that the quality is maintained throughout the study and will write the study publications.

If new or emerging safety information that affects the benefit/risk assessment of the clinical trial negatively arise, the trial will be discontinued.

The study will be monitored by the Regional Clinical Trial Unit at Oslo University Hospital and the Norwegian Medicines Agency may also want to conduct an inspection. The monitor will get access to source data.

### Adverse events

The sponsor-investigator will pay special attention to the following subset of adverse events (AEs) of special interest (AESIs) – similar as in (35):

a) Cardiac function that could be indicative of QT interval prolongation or cardiac arrhythmias: Torsade de pointes, sudden death, ventricular extrasystoles, ventricular tachycardia, ventricular fibrillation and flutter, syncope (non-postural), and seizures.
b) Suicide risk: suicides, suicide attempts, self-injurious behavior associated with suicidal ideation, or severe suicidal ideation (scores of 4 or 5 on the C-SSRS).
c) Abuse potential: AEs involving the terms of Behavioral addiction, Drug abuser, Substance abuser, Dependence, Intentional product misuse, Overdose (accidental, intentional, or prescribed), or Drug diversion in cases that are related to MDMA or “Ecstasy.”

### Investigational medicinal product (IMP)

This study will investigate the effects of two open-label MDMA Dosing Sessions of MDMA as described in the table below. Similar MDMA doses to those in the present study have been safely used in previous PTSD trials (28, 30). The dose regimen is shown in Table 4. The rationale of the supplemental dose is to increase the durability of the psychoactive effects.

**Table 4:** Dose regimen of MDMA.

**Dosing session**	**Initial dose**	Supplemental dose*	**Min-max cumulative dose**
1	80 mg	40 mg	80 mg to 120 mg
2	80 or 120 mg	40 or 60 mg	80 mg to 180 mg
**Total cumulative dose**			160 mg to 300 mg

*Unless tolerability issues emerge with the first dose or the participant declines.

### IMP management

A permit to prescribe MDMA has been obtained from The Norwegian Directorate of Health. MDMA has been supplied by the Multidisciplinary Association for Psychedelic Studies (MAPS). Manufacture and export was performed by SHARP CLINICAL SERVICES, INC., 2400 BAGLYOS CIR, BETHLEHEM, USA. Good Manufacturing Practice (GMP) has been maintained at all stages of manufacture. All IMP labels will comply with local and national regulations. MDMA will be handled and stored in accordance with all Norwegian regulations. The IMP will be stored and dispensed by the hospital's pharmacy. Study drug treatment will be dispensed at the dosing visit and unused treatments will be re-collected and recorded to verify drug accountability and compliance. At the completion of the study, all unused medicine will be destroyed in accordance with standard safety procedures.

### Long-term follow-up and qualitative interview

At least 3 months after completion of treatment, participants will be invited to a long-term follow-up (LTFU) visit. At the LTFU, a mixed method approach with quantitative and qualitative assessments is taken. We will repeat the same measures as in the outcome (MADRS and SDS) and study termination (BDI-II, EQ-5D-5L, AUDIT, DUDIT, SDS, WEMWBS, TAS-20, BIS, PCL-5, and GAD-7) visits. The LTFU visit also includes a semi-structured interview in which the participants will be invited to explore the perceived experiences with MDMA-AT for MDD. This interview will be transcribed and analyzed with the qualitative method Interpretative Phenomenological Analysis (IPA).

Including a qualitative approach will allow a greater focus on the perspective of the users. There is an insufficient number of qualitative studies exploring participant's experiences of MDMA-AT. To our knowledge, only one qualitative study has explored how participants experience different features of MDMA-AT (59). The main aim of this subproject will be to expand and add nuances to the quantitative measures. This will hopefully broaden our understanding of MDMA-AT for MDD. All included participants will be asked to participate in this qualitative study. A phenomenological/hermeneutic approach will be used to analyze the data. A semi-structured qualitative interview has been developed. This interview will cover the following themes: Experiences with depression, experiences with MDMA-AT, experiences of processes that were helpful and unhelpful, experiences with identity, and experiences with relations to others (family, friends, work, school etc.).

### Dissemination

The results of the study will be published in international peer reviewed journals. The results of the study would also be presented at scientific conferences and in the media.

## Conclusions

Here we have presented a trial protocol for a phase II, proof of principle trial assessing MDMA-AT as a treatment for MDD. This protocol's strength is that it will provide key information about the proof of concept (recruitment, retention and acceptability) in advance of a future definitive study while exploring the safety and the initial impression of treatment efficacy. This study's key limitation is its inability to inform definitive effectiveness of MDMA-AT for MDD, as this is a small, open label pilot trial.

## Ethics statement

The trial has been reviewed and approved by the Norwegian Medicines Agency and the Regional Committee for Medical and Health Research Ethics. All staff have undergone Good Clinical Practice (GCP) training. The study has been registered at the EU clinical trials register (EudraCT number 2021-000805-26). All study sessions will take place at the public hospital Østfold Hospital Trust. This clinical study was designed and shall be implemented and reported in accordance with the International Conference on Harmonization (ICH) Guidelines for Good Clinical Practice (GCP), with applicable local regulations (including European Directive 2001/20/EC, US Code of Federal Regulations Title 21) and with the ethical principles laid down in the Declaration of Helsinki.

## Author contributions

Study conceived by T-MK, IG, LS, and OA. Protocol written by T-MK with template from MAPS. Study documentation written by T-MK. Regulatory approvals obtained by T-MK and OA. Supervision provided by OA and JR. Statistical analysis plan by T-MK in collaboration with the hospital's statistician. Trial clinical team T-MK, IG, and LS. Input to study rationale by DR. All authors contributed to the article and approved the submitted version.

## Funding

This is an investigator-initiated trial funded by South-Eastern Norway Regional Health Authority (project number 2021007) with a partial contribution from MAPS. MDMA has been provided by MAPS.

## Conflict of interest

Author T-MK received lecture honorarium from Lundbeck. Author LS was employed by Awakn Life Sciences Inc. Author OA was a consultant for HealthLytix and has received speaker honorarium from Lundbeck and Sunovion. Author IG was an advisor for Solrise Life Sciences. Several authors are employed at institutions T-MK, IG, LS, and OA at Østfold Hospital; DR at the Charité, which have received research support from MAPS for an MDMA-AT trial for PTSD. Author DR is founding member and honorary Board Chair of MAPS Deutschland, a Germany-based non-profit research and educational organization associated with MAPS. The remaining author declares that the research was conducted in the absence of any commercial or financial relationships that could be construed as a potential conflict of interest.

## Publisher's note

All claims expressed in this article are solely those of the authors and do not necessarily represent those of their affiliated organizations, or those of the publisher, the editors and the reviewers. Any product that may be evaluated in this article, or claim that may be made by its manufacturer, is not guaranteed or endorsed by the publisher.
